# Photoacoustic imaging of hidden dental caries using visible–light diode laser

**DOI:** 10.1002/acm2.13935

**Published:** 2023-02-24

**Authors:** Fikhri Astina Tasmara, Rini Widyaningrum, Andreas Setiawan, Mitrayana Mitrayana

**Affiliations:** ^1^ Department of Physics, Faculty of Mathematics and Natural Sciences Universitas Gadjah Mada Yogyakarta Indonesia; ^2^ Department of Dentomaxillofacial Radiology, Faculty of Dentistry Universitas Gadjah Mada Yogyakarta Indonesia; ^3^ Department of Physics Kristen Satya Wacana University Salatiga Indonesia

**Keywords:** hidden caries, photoacoustic imaging, visible light

## Abstract

**Background:**

Hidden caries is a type of tooth decay that is difficult to identify through visual diagnosis because teeth with hidden caries appear normal on the tooth surface but are damaged underneath.

**Methods:**

A photoacoustic imaging system based on visible light using a diode laser with a wavelength of 532 nm was developed to detect hidden caries in teeth.

**Results:**

The results indicate that the average of acoustic intensity level for healthy teeth is −74.2 ± 0.1 dB, and the average of acoustic intensity range for teeth with hidden caries is −81.2 ± 0.5 dB. The intensity level for the caries area varies depending on the severity of caries.

**Conclusion:**

Based on the acoustic intensity level measured by the interaction of teeth with laser light, the photoacoustic imaging system in the study can accurately detect the presence of hidden caries and recognize the difference between caries teeth and healthy teeth. This research can be developed into a prototype of a simple device that makes it easy to operate in dental practice.

## 1 INTRODUCTION

Nowadays, a significant proportion of adults are impacted by dental caries. The Global Burden of Disease Study 2019 indicated that over 3.5 billion people worldwide suffer from oral disorders, with caries affecting permanent teeth being the most prevalent problem. Globally, it is reported that 2 billion adults have permanent tooth caries and 520 million children have primary tooth caries.^1^ Dental caries, often known as tooth decay, is a disease that damages the tooth structures. Dental caries is induced by bacteria in the mouth, which produce lactic acids that directly attack the enamel layer of the tooth surface.^2^, ^3^ Dental caries can easily be visually diagnosed by an expert in dental practices. However, a type of caries that are difficult to identify through visual diagnosis have been recognized, namely, hidden caries.^4^ Hidden caries appears normal or only slightly demineralized on enamel of tooth surface. In simple terms, teeth with hidden caries appear normal on the outside, but the tooth structure is damaged inside.^5^ Although hidden caries may be approached using radiography for detection, radiation from x‐rays have significant side effects. Therefore, it is necessary to develop a non‐ionized radiation method that allows the hidden caries lesion in the tooth to be diagnosed from its surface.^2^, ^5^

One method that has been investigated for dental imaging is photoacoustic imaging (PAI). PAI is a non‐invasive diagnostic imaging technology that combines optical and ultrasound (US) imaging techniques to utilize the photoacoustic effect.^5^, ^6^ Compared to other imaging methods, such as light transillumination,^7^, ^8^ quantitative light‐induced fluorescence,^8^, ^9^ optical coherence tomography,^8^ diffuse optical tomography, US imaging, and fluorescence molecular tomography, PAI produces higher contrast and spatial resolution.^10^

A photoacoustic imaging system works by exposing a laser pulse to the biological tissue to be imaged, potentially causing some of the laser energy to be absorbed and converted into heat. The absorption coefficient is affected by the tissue's endogenous chromophore content. These endogenous chromophores in teeth (such as water, hemoglobin, and melanin) contribute to laser energy absorption.^6^ Photoacoustic signals will be produced as a result of the continuous thermoelastic expansion caused by laser illumination, which will be recorded in the detector and reconstructed as a photoacoustic image in the computer.^11^

Previous studies have shown that photoacoustic images can be generated from light with various wavelengths, such as LEDs,^12^, ^13^ xenon lamps,^14^ and diode lasers.^6^, ^15^ The research by Allen and Beard,^12^ which was further developed by Zhu et al.,^13^ has paved the way for the development of visible light‐based PAI modalities. The advancement of PAI in recent years includes diode laser as an alternative light source for PAI system. As a light source in a PAI system, the diode laser is dependable, practical, durable, affordable, and easy to operate.^15^, ^16^

Several groups have reportedly applied the photoacoustic method for diagnosis of dental caries. Since the report of Li and Dewhurst^17^ on PAI techniques in dentistry using near‐infrared pulsed laser sources, studies of PAI for biological hard tissues has been widely developed. The results show that the PAI technique has a great opportunity to be developed as an imaging tool for detection of tooth decay.^17^ In addition, Sharkawy and Sherif^18^ used a photoacoustic system to differentiate healthy teeth and tooth decay. However, in this study, an Nd:YAG laser was used as a light source. The results show a significant difference in the graphs and values of the surface acoustic wave pulse in healthy and damaged teeth.^18^ Hughes et al.^19^ used a photoacoustic microscope, which is a combination of a photoacoustic system and microscope, to detect early‐stage dental caries.^19^ In the following year, Cheng et al.^20^ also developed photoacoustic tomography to detect early‐stage dental caries.^20^ In addition, Arabpou et al.^21^ simulated the detection of early‐stage dental caries using a PAI system with a 633 nm light source.^21^ Subsequent studies also showed that a high intensity of the PA signal could be observed in regions with lesions, whereas healthy surfaces showed much less PA signal in early caries detection.^22^, ^23^ The application of a PAI system to detect hidden caries has also been carried out by Koyama in 2017 and 2018 with a photoacoustic system using a fiber‐based imaging system.^5^, ^24^

Based on previous research, PAI systems have been used to detect caries; however, this research has been limited to the detection of open caries and has not discussed hidden caries in detail. Therefore, this study aims to investigate the detection of hidden caries using a PAI system with a visible light‐based diode laser as the light source. This study will compare photoacoustic image and acoustic intensity levels obtained from healthy teeth, teeth with caries, and teeth with hidden caries.

## 2 MATERIALS AND METHODS

### 2.1 Tooth sample preparation

The human teeth sample were donated by various individuals and patients without identifiers. Before PA imaging, all samples were fixed for 2 h in a 70% alcohol solution.

### 2.2 PAI system

**FIGURE 1:**
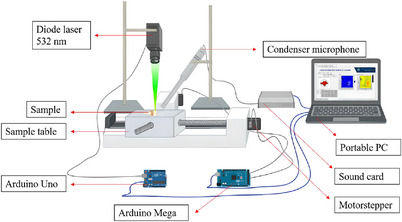
Photoacoustic imaging system utilized in the study.

Figure 1 illustrates the PAI system utilized for this study. As a light source, a laser diode with a 532 nm wavelength and 200 mW of maximum power was used. The photoacoustic signal was collected by the ECM8000 condenser microphone and transmitted to the computer via the sound card. A sample that has been placed on the sample table was moved along the XY axis by a stepper motor. The motor stepper movement and laser modulation are controlled via Arduino Mega and Arduino Uno microcontrollers.

### 2.3 PAI system calibration

Each component was characterized before a photoacoustic system was put into operation. This method aims to ensure that the PAI system in use was suitable and operationally ready to generate accurate data. To ensure system readiness, three separate calibrations were conducted: condenser microphone, laser power, and stepper motor calibrations. When all characterization results match with the theory, the PAI system was assumed ready for use.

### 2.4 Image reconstruction process

Image reconstruction was performed with three different tooth samples in this study. Each sample had previously been identified as having hidden caries. The scanning process involves illuminating the sample with a laser diode at a frequency of 20 kHz and a duty cycle of 50%; the laser exposure was chosen based on the results of a previous calibration. To ensure that the methods employed in this study were valid and reliable, data collection was repeated three times. Each sample received the same exposure treatment, with three repetitions. The acoustic signal intensity data displayed is the average of each sample's repetition.

LabVIEW in personal computer will display the acoustic signal that was obtained. The obtained image is considered raw data that will be processed and analyzed using Python and OriginPro.

## 3 RESULT

**FIGURE 2:**
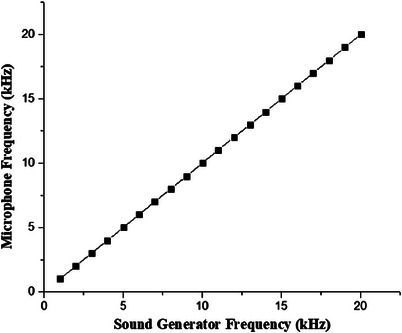
Correlation between the sound generator frequency and microphone frequency.

Figure 2 shows the results of the condenser microphone's characterization, which reveals a highly linear relationship between the sound generator frequency and measured frequency that was detected by condenser microphone in PAI system and displayed on LabVIEW which serves as the controller for the PAI system.

**FIGURE 3:**
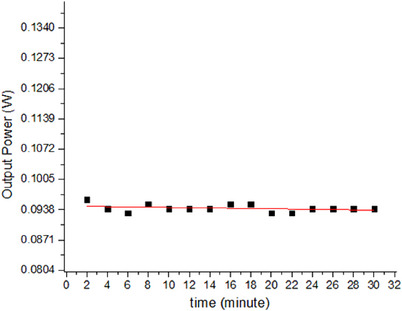
Characterization of the laser power (W) observed in respective interval time.

After choosing the optimal frequency for photoacoustic imaging in this study, the laser power stability was characterized. Figure 3 shows the results of the laser power output characterization. The stability of the laser power at a frequency of 20 kHz and duty cycle of 50% was measured for 30 min.

**FIGURE 4:**
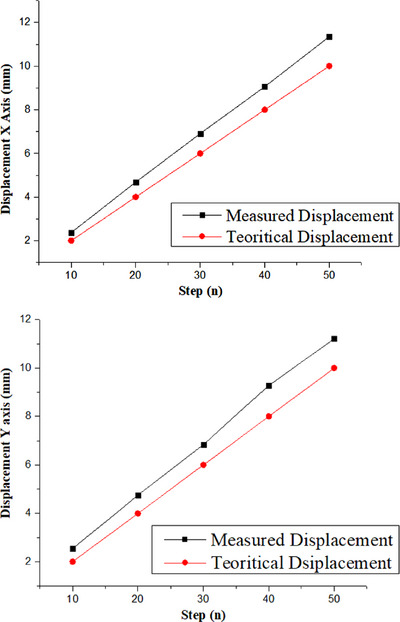
(a) Average shift of the x‐axis for each step; (b) average shift of the y‐axis for each step.

The characterization of the stepper motor was carried out on the movements of the x‐axis and y‐axis, respectively. The change in the position on the x‐axis is shown in Figure 4a, and the change in the position on the y‐axis is shown in Figure 4b. The black square symbol represents the measured shift, while the red circle represents the shift that should occur. Based on the graph obtained, the shift obtained is linear with the shift expected.

**FIGURE 5:**
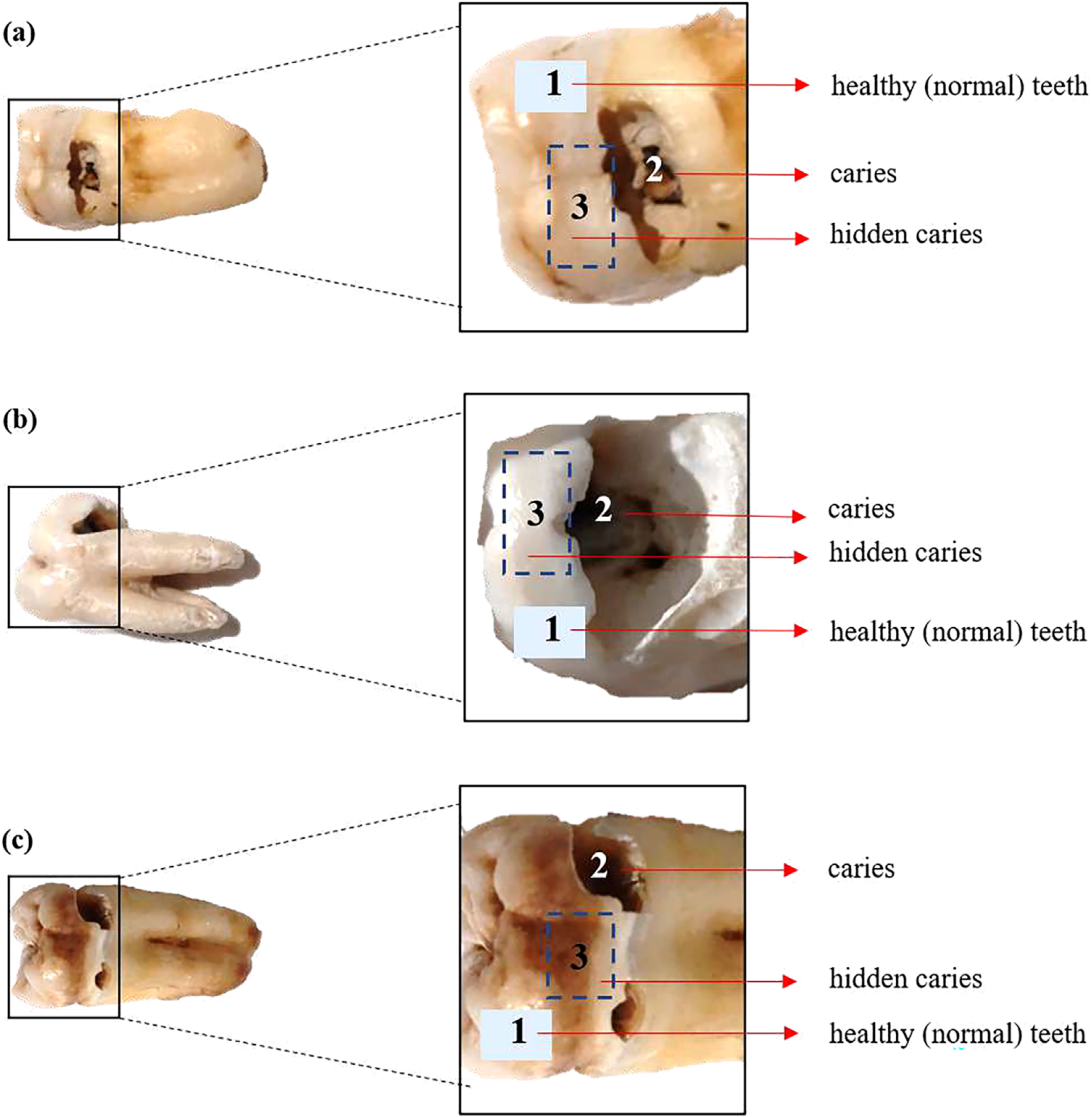
Samples of teeth with conditions indicative of caries and hidden caries. The region of (1) normal teeth, (2) caries, and (3) hidden caries are shown in the picture.

Previous studies have reported that the photoacoustic intensity level generated by each sample's surface conditions produced different results. In this study, the differences in the recorded photoacoustic signal intensity levels among healthy (normal) teeth, caries teeth, and hidden caries areas are shown. All teeth samples are shown in Figure 5.

Figure 5 shows the region of normal teeth, caries, and hidden caries. Healthy teeth region marked by number 1. Caries‐affected areas of teeth are visible in regions that have changes in surface shape, such as poaching and change in color to brown (marked by number 2). The area of the tooth with hidden caries is marked with a dashed blue line (number 3), in which the tooth enamel appears undamaged but the underlying structure is damaged.

**FIGURE 6:**
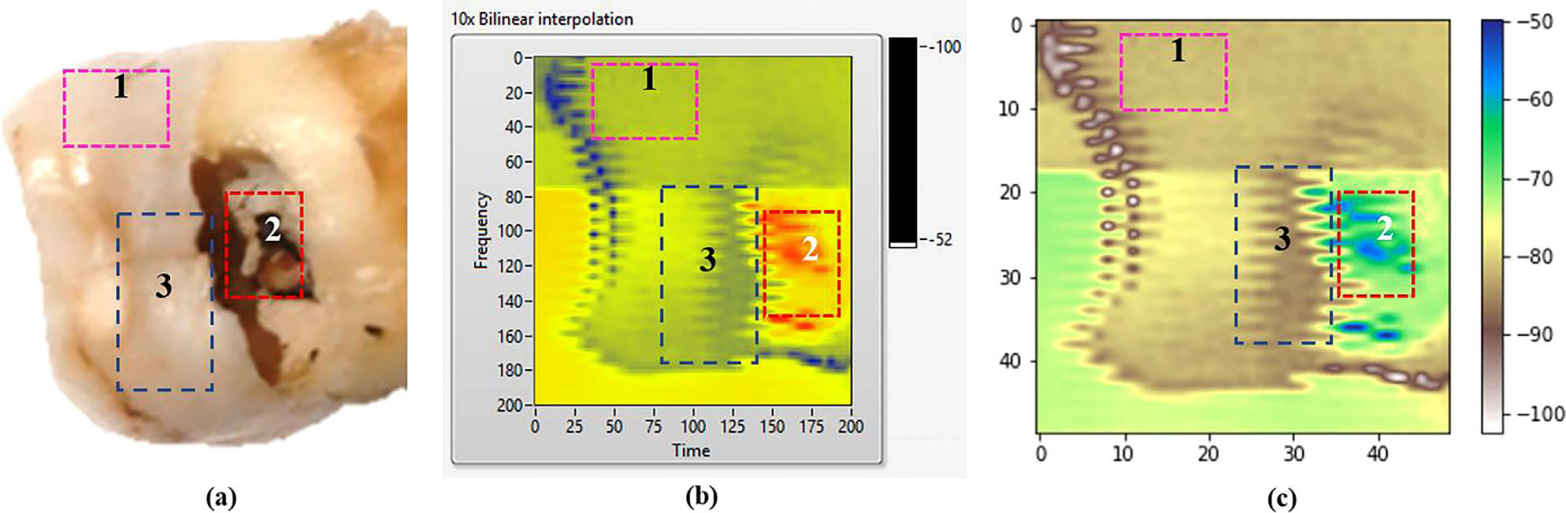
(a) tooth sample; (b) photoacoustic image displayed in LabVIEW; (c) photoacoustic image after processing in Python. Sample and photoacoustic image shows areas of normal teeth (1), caries (2), and hidden caries (3).

The result of photoacoustic scan was performed on each sample to determine the average intensity level of each tooth. Figure 6 shows the difference photoacoustic images in LabView and Python.

**FIGURE 7:**
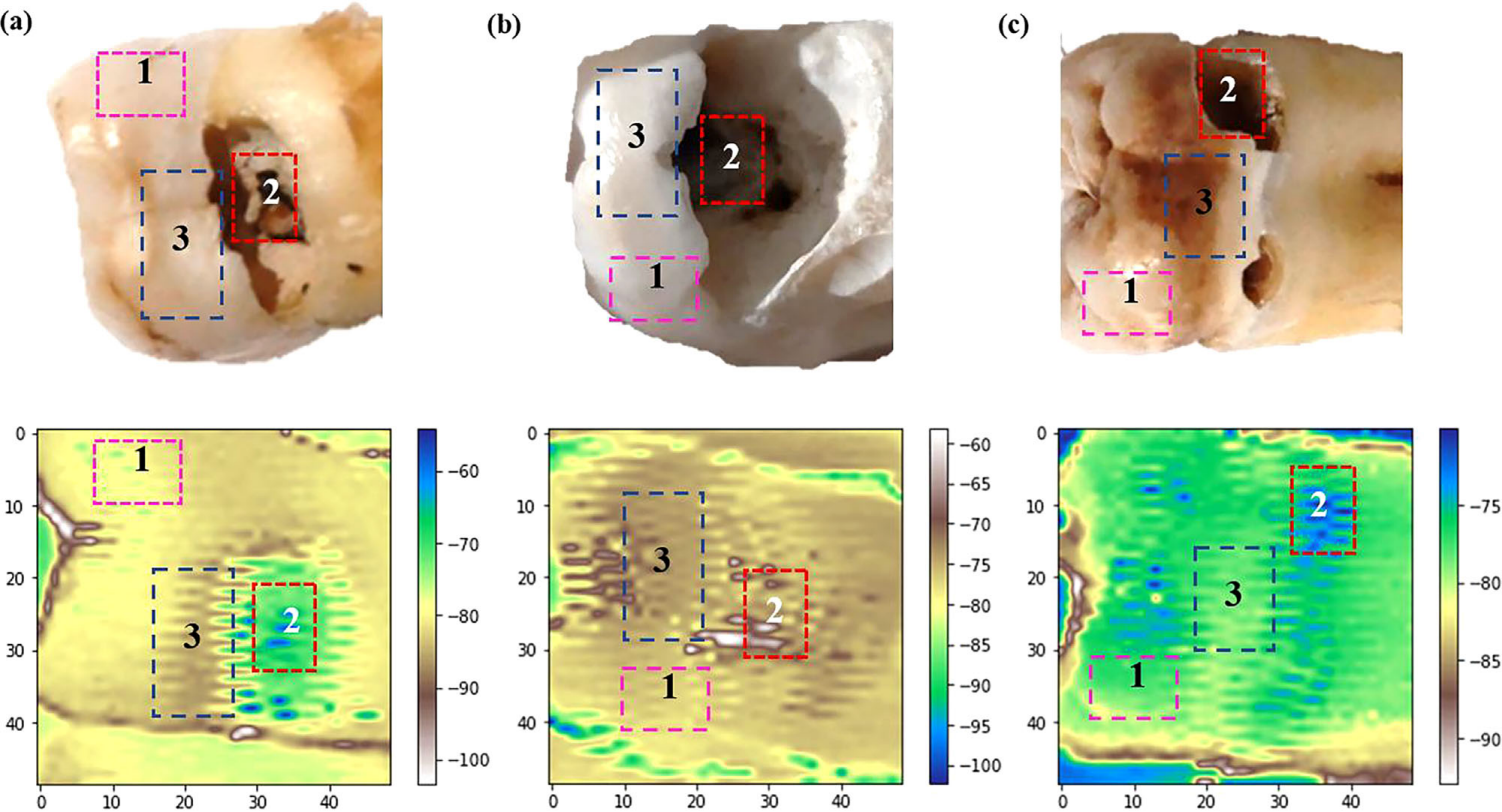
(a) First tooth sample; (b) second tooth sample; (c) third tooth sample. The images in the second line are photoacoustic images of each of the samples shown above. Sample and photoacoustic image shows areas of normal teeth (1), caries (2), and hidden caries (3).

Figure 7 shows the photoacoustic images for all tooth samples that have been processed by the Python program. In addition, the area of the tooth that is indicated to have hidden caries displays a different color than the surrounding healthy teeth in the photoacoustic image for each obtained sample. The intensity level of each image can be determined from the color scale to the right of the Python‐processed acoustic image.

**FIGURE 8:**
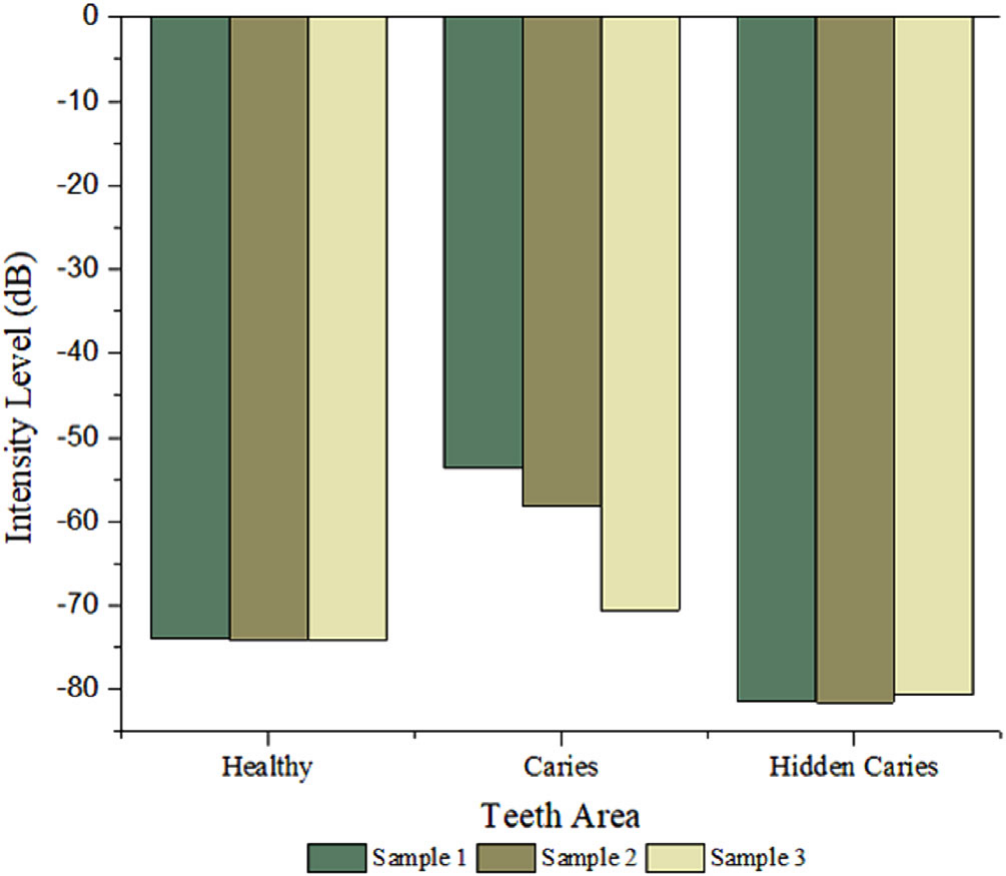
Acoustic intensity level of each tooth condition.

The obtained photoacoustic images have allowed us to demonstrate that the PAI system can represent and evaluate areas of healthy teeth and caries and identify hidden caries non‐invasively. Figure 8 shows the measured intensity level values of each tooth condition in several repeated measurements. The difference in the level of caries damage in each tooth causes the non‐uniformity of the intensity level measured in the caries area. The measured intensity level can be implemented as a standard for future measurements. Table 1 displays the results of each sample's intensity measurement.

**TABLE 1:** Average acoustic intensity level measured in each sample

**Teeth condition**	**Intensity level (dB)**
Healthy	−74.2 ± 0.1
Hidden caries	−81.2 ± 0.5
Caries	Depending on the degree of caries damage

## 4 DISCUSSION

In this study, a new application of a PAI system in dentistry was developed, which can detect the presence of hidden caries and indicate the severity of open caries based on the difference in the measured acoustic intensity level. In previous studies, the detection of hidden caries based on non‐visible light using a near‐infrared light source at 780 nm has shown promising results.^22^, ^25^ Therefore, the development was carried out on a PAI system using a visible light source in the form of a laser diode with a wavelength of 532 nm to produces acoustic waves when interacting with the sample.

The produced photoacoustic signal is equal to the light absorption by soft and hard tissues. Therefore, a direct relationship exists between the increase in the photoacoustic signal, tooth tissue and surface area.^22^, ^26^ In a previous study by da Silva et al.,^22^ it was confirmed that each tooth showed a consistent increase in photoacoustic signal depending on the condition.^22^ Although in some studies, visible light wavelengths (400–700 nm) are limited by strong light scattering in dental tissue, making it challenging to imaging through tooth structure thicknesses of 1–2 mm^7^. In this study, a visible light‐based PAI system was used to detect hidden caries that are difficult to detect using visual analysis in clinical examination.

The effectiveness of a photoacoustic system is dependent on the sensitivity of each of its components, which has a significant impact on the generation of the strongest acoustic signal. In PAI system, microphone sensitivity, laser power, and sample table movement play significant roles.^15^, ^27^ The results of the principal component characterization are depicted in Figures 2, 3, 4. Figure 2 shows a very clear linearity between the given modulation frequency and measured frequency by the condenser microphone. This linearity relationship makes sure that the acoustic signal captured by the condenser microphone is stable and corresponds to the frequency generated by the sample's interaction with light.

Figure 3 shows the relationship between the laser output power and exposure time with a 50% duty cycle. After 30 min, the laser's output power was found to be stable between 0.093 W and 0.096 W. The laser output power almost did not change during the scanning time, so the laser exposure was in a stable state during the photoacoustic imaging scanning process.

The shift stability of the sample table is shown in Figure 4. The x‐axis and y‐axis displacements are measured by comparing the expected step to the measured step. On the x‐axis, the largest difference between the expected shift and measured shift is 1.32 mm, whereas the largest difference between the expected shift and measured shift on the y‐axis is 1.2 mm. Based on these results, the step suitability of the step motor shift ensures that no sample area is missed or double‐scanned during the photoacoustic scanning process.

After characterization, the suitability of each component produced a clear image; in this case, it can diagnose hidden caries even with wavelengths in the visible‐light region. Figures 6 and 7 illustrate the photoacoustic image results for each sample. This study's sample was restricted to differences in the severity of caries.

In Figure 6b, the image displayed in LabVIEW displays different intensity levels based on the resulting color. Areas of healthy teeth are shown in lighter green (pointed by number 1). The area of the exposed caries tooth is very clearly imaged, marked with an orange to red color (pointed by number 2) depending on the severity of caries that occur. Whereas those of teeth indicated by hidden caries are shown in dark green (pointed by number 3). The processed photoacoustic image is visualized in Figure 6c, as a result of the Python program. The image processing that is performed will not change the original image; it will only increase contrast and clarify the boundaries between tooth regions. The intensity level is displayed on the scale bar to the image's right.

Similar results are also shown in Figure 7 for the all three samples. Based on the tested samples, the second sample had the most severe level of caries, and the third sample showed the least caries based on its intensity level. In the second sample (Figure 7b), the hidden caries region is shown in dark red (pointed by number 3), whereas for the third sample, the hidden caries region is shown in green, which is lighter than the other regions (pointed by number 3). The healthy area is always pointed by number 1, and the caries area is pointed by number 2.

As the color produced by each sample is different, despite the fact that it can still provide a clear image, a factual indicator is still required. Therefore, Figure 8 and Table 1 reveal the intensity level measurement based on the acoustic signal.

The results obtained from measuring the average intensity level of each sample tend to be uniform in Table 1. Based on the results, the area of healthy teeth has a lower intensity level than the area of teeth affected by caries. This is because bacteria in the mouth produce lactic acid, which damages tooth enamel. The excessive production of lactic acid will progressively destroy tooth enamel from the inside and outside. When the tooth enamel is damaged from the outside, tooth structure changes present irregularly shaped holes and affect the acoustic intensity produced when a sample interacts with the laser beam. The obtained results are consistent with previous research studies that the intensity of the resulting acoustic signal increases proportionally with the severity/damage level of the tooth structure.^27^, ^28^ Image contrasts are impacted by variations in the average acoustic intensity level. Because the acoustic intensity level of caries differs, the value of the intensity level obtained in the area of caries is different for each tooth.

In the case of hidden caries, the tooth's chromophore probably decreases, which leads to a reduction in the absorbance coefficient value. Reduced absorbance coefficient in the tooth area affects the intensity value of the resulting acoustic signal, causing the acoustic intensity level to be lower than in normal teeth. When the sample is irradiated with a laser beam, the light that penetrates the outermost layer of tooth enamel does not interact optimally with the internal structure because the tooth structure in hidden caries has been damaged. Due to this suboptimal interaction, the produced intensity level is lower than the intensity level in the healthy tooth area.

This study demonstrates the viability of a PAI system for detecting hidden caries. The results of this study are consistent with previous studies employing a PAI system to examine dental caries.^22^, ^29^ This research is still limited to ex vivo study and further researches are needed to compare the photoacoustic imaging with other modalities for hidden caries detection.

## 5 CONCLUSION

A PAI system based on visible light using a diode laser with a wavelength of 532 nm was developed to detect the presence of hidden caries in teeth. Based on the acoustic intensity level measured by the interaction of teeth with laser light, this system can accurately detect the presence of hidden caries and recognize the difference between caries and healthy teeth. The results indicated that the average intensity level for healthy teeth was −74.2 ± 0.1 dB, whereas the intensity range for teeth with hidden caries was −81.2 ± 0.5 dB. The intensity level for the caries area varied depending on the severity of caries. However, further studies must be conducted to develop the hardware and software components of the PAI system.

## AUTHOR CONTRIBUTIONS

**Fikhri. A Tasmara**: data collection, formal analysis, writing‐original draft preparation. **Rini Widyaningrum**: conceptualization, result validation, reviewing and editing. **Andreas Setiawan**: reviewing. **Mitrayana**: conceptualization, supervision, result validation, reviewing. All authors discussed the result and commented on the manuscript.

## CONFLICT OF INTEREST

The authors declare no conflicts of interest.

## Data Availability

The authors confirm that all data required to support the findings described above are available within the article.
